# Major epidemiological features of first‐ever ischemic stroke in Tuzla Canton, Bosnia and Herzegovina

**DOI:** 10.1002/hsr2.445

**Published:** 2021-11-29

**Authors:** Adnan Mujanovic, Dzevdet Smajlovic

**Affiliations:** ^1^ Medical Faculty University of Tuzla Tuzla Bosnia and Herzegovina; ^2^ Department of Neurology University Clinical Center Tuzla Tuzla Bosnia and Herzegovina

**Keywords:** Bosnia and Herzegovina, epidemiology, first‐ever, incidence, ischemic stroke, risk factors

## Abstract

**Background and aims:**

Opacity of data on stroke for Bosnia and Herzegovina (B&H) is mainly due to the lack of a unified national stroke registry. This article aims to present updated epidemiological data on the etiology and risk factors for first‐ever ischemic stroke in Tuzla Canton, B&H.

**Methods:**

This retrospective hospital‐based study included all first‐ever ischemic stroke patients admitted between January 1, 2018 and December 31, 2018 at the Neurology Department, University Clinical Center Tuzla.

**Results:**

First‐ever ischemic stroke was diagnosed in 739 patients. Leading risk factors were hypertension (94%), diabetes mellitus (40.7%), and dyslipidemia (38.8%). The most common stroke subtypes were atherothrombotic (36.8%), cardioembolic (21.9%), and stroke of undetermined etiologies (19.2%). Mean NIHSS score at discharge was 13 (IQR 2‐16), and favorable patient outcome (mRs ≤2) was recorded in 26.4% patients. Men (aOR 0.39; 95% CI 0.24‐0.64) and younger patients (aOR 0.96; 95% CI 0.93‐0.98) had significantly higher probability of having a favorable outcome at discharge. Dyslipidemia could be considered as a predictive factor for patient outcome (aOR 0.66; 95% CI 0.43‐1.00).

**Conclusions:**

More than 92% of our patients had at least one modifiable risk factor, with hypertension and diabetes being at the forefront. One out of four patients had become functionally independent at discharge, while hospital mortality was lower than in other Eastern European countries. The overarching goal should be steered toward the development of a national stroke registry, which should be used as a reference for all further stroke management activities.

## 1 INTRODUCTION

Ischemic stroke (IS) is defined as a multifactorial heterogeneous disorder, characterized by a sudden symptom onset, which can be directly correlated to the brain's injury site. IS has undergone further etiological categorization according to the TOAST trials.^1^ Stroke contributes considerably toward long‐term disability with non‐negligible emotional and socioeconomic repercussions for patients, their families, and healthcare providers. In 2017, a total of 9 million people across Europe were living with stroke, with total disability‐related costs amounting to over €60 billion.^2^ In the same year, stroke was responsible for over 438 000 deaths across the European Continent. Adjusted stroke‐related health and social costs amounted to €3483 per stroke patient.^2^ Overpopulation and prolonged life expectancy have significantly increased stroke incidence rates.^3^ Substantial geographic and regional differences of stroke incidence have contributed toward increased burden of this disease in low‐ and middle‐income countries, making it a serious public health issue.^4^ In 2020, it was estimated that stroke and cardiovascular disease were the leading causes of lost healthy life‐years.^5^ Opacity of stroke data for Bosnia and Herzegovina^6^, ^7^, ^8^ (B&H) is mainly due to a lack of a national stroke registry. The last recorded efforts to present updated stroke data were made in 2014, but again this could not be replicated at the country level.^9^ Neighboring countries offer regular updates on stroke incidence and prevalence and plan their public health policies accordingly.^10^, ^11^, ^12^, ^13^ Even though lower stroke mortality rates have been observed lately,^14^ IS ranks high in both incidence and disability throughout our region.^9^, ^13^ This article aims to present an up‐to‐date overview on the epidemiology, etiology, risk factors, and mortality for first‐ever ischemic stroke (FEIS) victims in Tuzla Canton, B&H.

## 2 MATERIALS AND METHODS

World Health Organization's definition was used for the purpose of this study, with IS being defined as “rapidly developing symptoms and/or signs of focal, and at times global, loss of cerebral function, with symptoms lasting more than 24 hours or leading to death with no apparent cause other than that of vascular origin.”^15^ TOAST criteria have been applied for IS etiology clasification.^1^ All patients gave informed consent for their data to be used in this study. In case the patient had a loss, or impairment, of consciousness, consent was obtained from either the accompanying care giver or a family member during patients' stay in the Stroke Unit, following recent recommendations for consent acquirement in acute scenarios.^16^ The study was approved by the Research Ethics Committee of the University Clinical Center Tuzla (Ethics Committee approval number 07‐18‐66/19) and was performed in accordance with the ethical standards of the 1964 Declaration of Helsinki and its later amendments.

### 2.1 Data collection and screening

**FIGURE 1:**
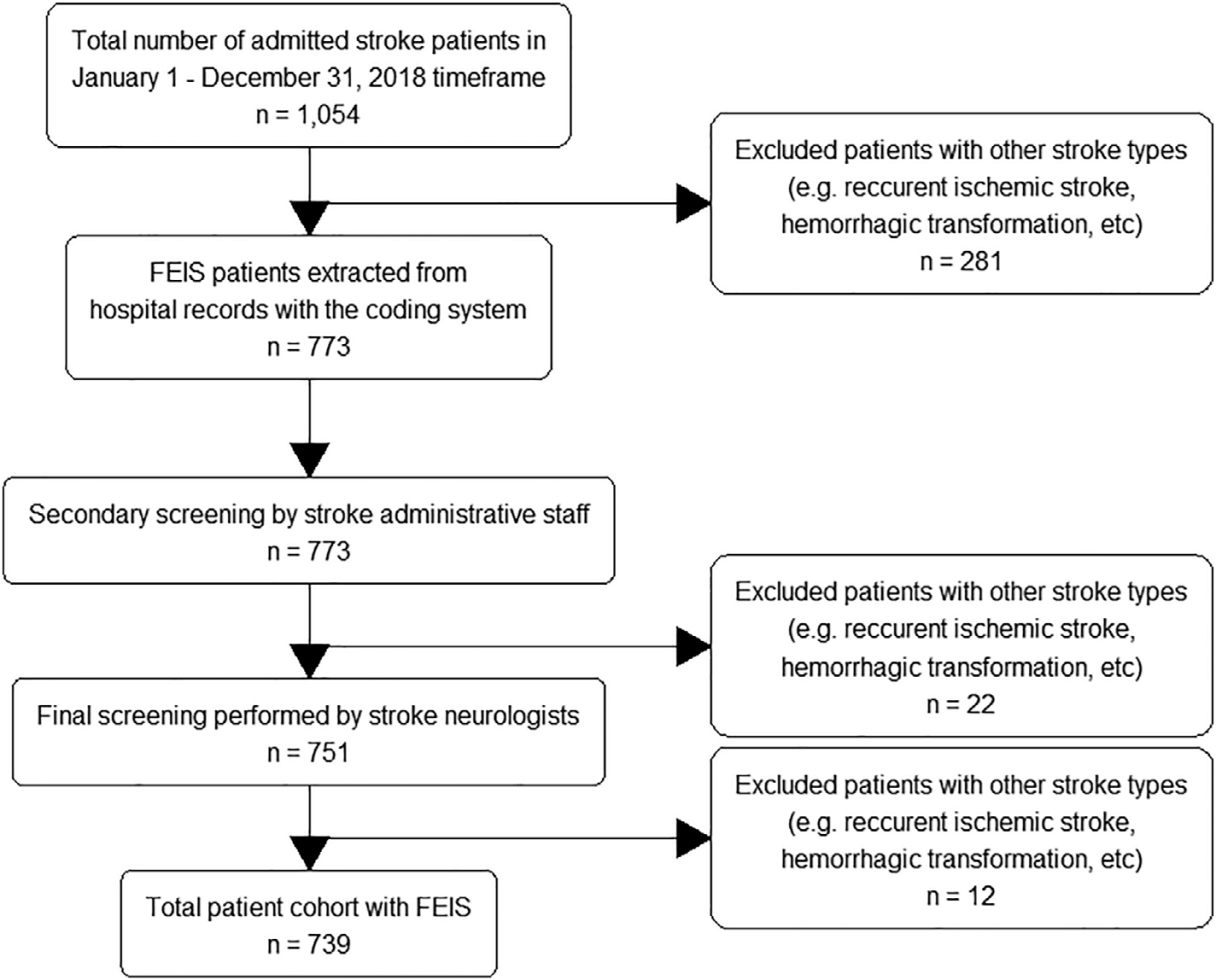
Study flowchart

A retrospective analysis of hospital‐based patient registry for FEIS admitted at the Stroke Unit, Neurology Department, University Clinical Centre Tuzla between January 1, 2018 and December 31, 2018 was performed. During this period, the number of admitted stroke patients was n = 1054. Initially, all FEIS cases were extracted using the hospital administrative coding system (n = 773). All other types of strokes were excluded, including recurrent ischemic strokes, hemorrhagic transformations of ischemic strokes, hemorrhagic strokes, and so on. Afterward, every single case was screened by two administrative staff assistants, who had been previously trained and have several years of experience in managing stroke patients' paperwork. Lastly, two stroke neurologists performed the final screening and re‐evaluation of each individual case, upon which the final patient cohort constituted n = 739 patients. Flowchart details are presented in Figure 1. This three‐step process ensured better compliance of data reliability because administrative stroke coding alone has proven to be unreliable in some cases.^17^

### 2.2 Study population

Population characteristics were taken from the registry of the Federal Institute for Statistics,^18^ where, according to their age, citizens of Tuzla Canton were already separated into three categories: (I) 0 to 14; (II) 15 to 64; (III) 65+. For the purpose of this study, and in order to comply with the Federal Institute's already present stratification listing, the cutoff age for calculating hospital stroke incidence was 14 years. Considering this, total population of the Tuzla Canton region comprised 375 913 (182 070 men and 193 843 women) citizens.

### 2.3 Clinical and laboratory parameters

The following clinical and laboratory parameters were assessed: hypertension, heart disease, atrial fibrillation, diabetes mellitus, dyslipidemia, active smoking status, alcohol overuse, and positive family history. Hypertension was defined as a systolic blood pressure of >140 mmHg, diastolic blood pressure of >90 mmHg, or previously documented hypertension treatment.^19^ Heart disease history included a previously confirmed diagnosis of at least one of the following: angina pectoris, myocardial infarction, cardiomyopathy, heart failure, atrial fibrillation, and other heart rhythm disorders. Diabetes mellitus was present when the fasting blood glucose concentration exceeded 7.0 mmol/L and/or plasma glucose 11 mmol/L at any time of day, or when there was a documented use of a blood‐sugar‐lowering drug before stroke onset.^20^ Dyslipidemia was defined based on the levels of total serum cholesterol (>5.0 mmol/L), low density lipoproteins (>3.0 mmol/L), and triglycerides (>2.0 mmol/L).^21^ Active smoking status was noted in case of smoking 10 cigarettes/day 6 months prior to stroke onset, and was considered absent in case the patient had never smoked or had stopped smoking for at least 1 year prior to stroke occurrence.^22^, ^23^ Alcohol overuse indicated a consumption of >100 mL alcohol/day during the past 2 months, or acute alcohol intoxication within a 24‐hours cycle of stroke onset.^23^, ^24^ Positive family history included either of the following: (a) one first‐degree relative with early‐ (<65 years) or late‐ (≥65 years) IS onset, or (b) at least one second‐degree relative with early‐ or late‐ IS onset.^25^ All data were extracted from the patients' hospital records.

### 2.4 Examination and imaging

IS was confirmed by neurological examination, neuroimaging methods, and laboratory tests. Final stroke diagnosis was always re‐evaluated by a stroke neurologist or an attending senior neurologist. Neurological examination focused on initial screening of vital functions (airway, breathing, circulation monitoring), as well as on the consciousness level, head and gaze deviation, and presence of movement lateralization. Neurological deficit severity on admission and discharge were assessed by the National Institutes of Health Stroke Scale (NIHSS).^26^ Vital status and independent functional outcomes were assessed by the modified Rankin Scale (mRS) across two different time points: upon admission, and 1 month after stroke onset.^27^ Primary neuroimaging method was a non‐contrast head computed tomography (CT) scan, due to its sensitivity and time effectiveness for hemorrhagic stroke exclusion. Magnetic resonance imaging (MRI) was rarely used because of its limited availability, time‐saving measures, and MRI eligibility criteria.^28^

### 2.5 Statistical analysis

Prespecified analysis was performed in 739 patients with FEIS, comparing baseline, clinical, and outcome variables. The Shapiro–Wilk test was used to evaluate variable distribution. Non‐normally distributed continuous variables were expressed as median and interquartile range (IQR), and categorical variables as frequencies and percentages (%, n/N). Chi‐square or Fisher's exact test was used to compare categorical variables and independent *t*‐test or Mann–Whitney *U*‐test for continuous variables. Multivariable logistic regression was adjusted for confounders and used to assess associations for favorable patient outcome. Prespecified confounders were sex (binary categorical), age (continuous), hypertension (binary categorical), diabetes (binary categorical), dyslipidemia (binary categorical), smoking (binary categorical), alcohol overuse (binary categorical), family history (categorical), NIHSS on admission (continuous variable, aOR referring to 1‐point increase), mRS on admission (continuous variable, aOR referring to 1‐point increase), and the use of thrombolysis (binary categorical). Outputs of regression analyses are presented as adjusted odds ratios (aOR) and corresponding 95% CIs. SPSS software for Windows (version 20, SPSS Inc., Chicago, Illinois) was used for the statistical analysis of the data. *P* < .05 on a two‐sided test was considered to be statistically significant.

## 3 RESULTS

Between January 1, 2018 and December 31, 2018, 739 patients were diagnosed with FEIS, of whom 48.4% (358/739) were men and 51.6% (381/739) women. Mean age of the group was 72.3 (IQR 65‐80), or, when stratified by sex, 71 (IQR 64‐79) and 73.6 (IQR 67‐92) for men and women, respectively. The hospital incidence rate of 1.96/1000 (men = 0.95; women = 1.01) was calculated with data from the recent population registry.^18^

The most common stoke risk factors were hypertension (94.1%, n = 695/739), diabetes mellitus (40.7%, n = 301/739), and dyslipidemia (38.8%, n = 287/739). Diabetes mellitus was significantly more present in women (*P* < .05; aOR 1.41 [95% CI 1.00‐1.98]), while smoking (*P* < .001; aOR 0.39 [95% CI 0.28‐0.59]) and alcohol overuse (*P* < .001; aOR 0.03 [95% CI 0.01‐0.11]) were mostly associated with men, as shown in Table 1.

**TABLE 1:** Stroke risk factors

	Men (n = 358)		Women (n = 381)		Total (n = 739)		aOR (95% CI)	*P*‐value
	N	%	N	%	N	%		
Hypertension	332	92.7	363	95.3	695	94.0	1.35 (0.62‐1.42)	.42
Angina	48	13.4	40	10.5	88	11.9	0.64 (0.44‐1.13)	.08
Cardiomyopathy	71	19.4	103	27.0	174	23.5	1.44 (0.84‐1.86)	.09
Atrial fibrillation	58	16.2	95	24.9	153	20.7	1.03 (0.88‐1.17)	.94
Other heart rhythm disorders	20	5.6	19	4.9	39	5.3	1.28 (0.95‐1.72)	.27
Diabetes mellitus	127	35.5	174	45.7	301	40.7	1.41 (1.00‐1.98)	.05*
Dyslipidemia	150	41.9	137	35.9	287	38.8	0.95 (0.36‐1.80)	.77
Smoking	132	36.9	57	14.9	189	25.6	0.39 (0.28‐0.59)	.001***
Alcohol overuse	76	21.2	3	0.8	79	10.7	0.03 (0.01‐0.11)	.001***
Positive family history	120	33.5	121	31.7	241	32.6	1.13 (0.59‐2.14)	.49

Abbreviations: aOR, adjusted odds ratio; CI, confidence interval.

* Correlation significant at the 0.05 level (two‐tailed);

*** Correlation significant at the 0.001 level (two‐tailed).

The most common IS subtypes were atherothrombotic (36.8%, n = 272/739), cardioembolic (21.9%, n = 162/739), and stroke of undetermined etiologies (19.1%, n = 141/739). Distribution of IS subtypes was without statistical significance between the sexes (Table 2). Neuroimaging was performed mostly with a non‐contrast head CT (97.7%, n = 722/739), while MRI was used only in individual cases (9.5%, n = 70/739).

**TABLE 2:** Ischemic stroke classification and imaging

	Men (n = 358)		Women (n = 381)		Total (n = 739)		*P*‐value
	N	%	N	%	N	%	
Ischemic stroke subtypes							
Atherothrombotic	165	46.1	107	28.1	272	36.8	1.000
Cardioembolic	68	18.9	94	24.7	162	21.9	1.000
Lacunar	52	14.5	83	21.8	135	18.3	1.000
Other determined etiology	15	4.2	14	3.7	29	3.9	1.000
Undetermined etiology	58	16.2	83	21.8	141	19.1	1.000
Neuroimaging methods							
CT	353	98.6	369	96.9	722	97.9	.31
MRI	41	11.5	29	7.6	70	9.5	.28

Abbreviations: CT, computed tomography; MRI, magnetic resonance imaging.

Stroke severity was recorded for all 739 patients. Mean NIHSS score at admission was 11 (IQR 5‐16), and NIHSS score at discharge was 13 (IQR 2‐16). This difference was found to be significant, *t*(738) = −3.75, *P* < .001. Lower NIHSS score at discharge (NIHSS ≤8) was significantly more among men (*P* < .001; aOR 1.03 [95% CI 1.01‐1.05]), as shown in Table 3. Mean mRS score at admission was 4 (IQR 3‐5) and at discharge 3 (IQR 2‐5). In 20.9% (n = 155/739) of patients, a fatal outcome was registered. Although mortality was higher among women, the difference in mortality between the sexes was not statically significant.

**TABLE 3:** NIHSS and mRS scores

Outcome	Men (n = 358)		Women (n = 381)		Total (n = 739)		aOR (95% CI)	*P*‐value
	N	%	N	%	N	%		
NIHSS at admission	358	100.0	381	100.0	739	100.0	0.99 (0.40‐2.83)	.27
NIHSS at discharge	358	100.0	381	100.0	739	100.0	1.03 (1.01‐1.05)	.001***
mRS at admission	355	99.2	380	99.7	735	99.5	1.13 (0.33‐1.63)	.30
mRS at discharge	358	100.0	381	100.0	739	100.0	0.65 (0.41‐0.78)	.001***

Abbreviations: aOR, adjusted odds rato; CI, confidence interval; NIHSS, National Institutes of Health Stroke Scale; mRS, modified Rankin Scale.

^***^ Correlation significant at the .001 level (two‐tailed).

Different factors affected favorable patient outcome (mRS ≤ 2) at discharge, as shown in Table 4. Both sex and age played a role in patient outcome, with men (*P* < .001; aOR 0.39 [95% CI 0.24‐0.64]) and younger patients (*P* < .001; aOR 0.96 [95% CI 0.93‐0.98]) having significantly higher probability of achieving it. Expectedly, patients who had lower NIHSS score (*P* < .001; aOR 0.89 [95% CI 0.84‐0.95]) and lower mRS score at admission (*P* < .001; aOR 0.31 [95% CI 0.22‐0.44]) were more likely to have favorable outcome at discharge. Dyslipidemia was also found to be a predictive, though not statistically significant, factor for patient outcome (*P* < .052; aOR 0.66 [95% CI 0.43‐1.00]).

**TABLE 4:** Factors influencing favorable patient outcome (mRS ≤ 2)

	aOR	95% CI	*P*‐value
Sex	0.39	0.24‐0.64	.001***
Age	0.96	0.93‐0.98	.001***
Hypertension	1.27	0.47‐3.41	.634
Diabetes	1.08	0.68‐1.72	.737
Dyslipidemia	0.66	0.43‐1.00	.052
Smoking	1.53	0.90‐2.59	.114
Alcohol overuse	1.42	0.70‐2.89	.329
Family history	1.00	0.62‐1.60	.994
NIHSS on admission	0.89	0.84‐0.95	.001***
mRS admission	0.31	0.22‐0.44	.001***
Thrombolysis	0.44	0.13‐1.52	.196

Abbreviations: aOR, adjusted odds rato; CI, confidence interval; NIHSS, National Institutes of Health Stroke Scale; mRS, modified Rankin Scale.

*** Correlation significant at the .001 level (two‐tailed).

## 4 DISCUSSION

This is the first study that gives a thorough overview of FEIS epidemiological data in the area of Tuzla Canton after the last population census conducted in 2013. IS patients constitute two‐thirds of all admitted strokes in this Department, with higher prevalence among women, as previously demonstrated.^7^ A Croatian study group reported stroke as the second leading cause of death and as one of the most frequent cardiovascular diseases.^10^ Similarly, in Serbia stroke is the primary cause of mortality in women and secondary cause among men.^12^ Our results of age at stroke onset correspond to regional findings by the Croatian and Serbian study group.^11^, ^13^ This is also in accordance with several global studies which have confirmed the relationship between stroke onset and age and its impact on global stroke burden both in regard to high mortality and disability.^29^

Most stroke risk factors are alterable, providing the possibility of stroke‐risk management. Hypertension, diabetes, and dyslipidemia have all been previously reported as important factors for stroke onset.^30^ The risk factors most accountable for stroke incidence in Serbia were smoking, physical inactivity, hypertension, and obesity.^13^ We observed similar effects with hypertension and diabetes. High prevalence of hypertension in our study group might be due to unhealthy lifestyle choices, dietary regiments, obesity, lack of physical activity, active smoking status, and constant stress exposure. This is further supported by the latest hypertension study in B&H.^31^ While the presence of hypertension and atrial fibrillation was insignificant between men and women, the Croatian study group conversely reported statistically more frequent presence of these factors in women.^11^ Otherwise, the presented stroke risk factors in our study cohort conform to the reports by other European study groups^32^, ^33^ and fit with the delineated cardiovascular risk in middle‐income countries.^14^ Active smoker status was higher in the Croatian study group when compared to ours (32.3% vs 25.6%, respectively), while alcohol overuse was lower (8.6% vs 10.7%, respectively),^11^ given smoking and alcohol overuse were significantly more associated with men in both studies.^11^

General stress often accounts for hypertension, especially considering the already established link between anxiety and hypertension occurence.^34^ Moreover, IS events are increasingly affecting younger people, with a predicted twofold incidence increase during the coming years.^35^ This may be due to poor diet, obesity, hyperlipidemia, and sedentary lifestyle, all of which may lead to increased morbidity of several vascular diseases besides stroke. More than 92% (n = 680/739) of our patients had at least one modifiable stroke risk factor, which further places emphasizes on primary prevention not only for IS but also for other cardiovascular diseases. Developing healthy nutritional behaviors and habits should be one of the aims of future public educational campaigns. Additionally, health benefits of regular physical activity and exercise should not be neglected because of their role in prevention, or even reduction, of chronic noncommunicable diseases.^36^

Conversely, factors such as age, sex, race, and genetic makeup cannot be changed, warranting high‐risk patients to have regular checkups and consultations with their attending physicians. Systemic control of modifiable stroke risk factors, accompanied by established dietary and exercise habits, can be crucial for these high‐risk subgroups.

IS subtype analysis showed no significant statistical adherence between the sexes. Even so, atherothrombotic subtype was more common in men, both in the Croatian study group and ours.^11^ This finding is also consistent with previous studies carried out in this Department.^37^ Inversely, we have found that women are affected more by the lacunar subtype, instead of the cardioembolic ones, as has been previously reported.^38^ Stroke of undetermined etiology was registered among 19.1% (n = 141/739) of patients in our cohort, which is in accordance with the results of the systematic review reported by Hart et al.^39^

In the present study, 26.4% (n = 195/739) of patients became functionally independent at discharge (mRS ≤2), which is below the figure reported in a Japanese hospital‐based study by Yoenda et al^40^ and in a Canadian study by Yu et al.^41^ It is important to note that the average patient stay during their first hospitalization after FEIS is 8 to 9 days in our Stroke Unit. This should be strongly considered while interpreting these results and should serve as an additional explanation of the reported percentages. The median mRS value on discharge was 3, which is accordance with a Polish hospital‐based study.^42^ Hospital mortality of 20.1% (n = 155/739) is lower than reported for other Eastern European countries.^43^ Non‐favorable stroke outcome (mRS >2; NIHSS ≥20) was significantly more among women, which is consistent with other studies.^44^ Further actions should address facilitation of a well‐defined stroke protocol that can provide comprehensive in‐ and out‐patient management, especially for patients with non‐favorable stroke outcomes.

This study has several limitations. First, there is a lack of availabity of complete patient data (eg, previously used medications). Second, because of the clinical presentation and outcome of some patients, not all diagnostic protocols could be performed. Likewise, we did not take into consideration the comparison of intravenous thrombolysis and mechanical thrombectomy because that data is beyond the primary epidemiological scopes of this paper. Aside our clinical center, there is also one other secondary‐level community hospital in Tuzla Canton that admits stroke patients; therefore further studies should also consider including these patients. Lastly, this study was a single institution‐based analysis, limiting the generalizability of its results and warranting a multi‐centric study throughout clinical centers in B&H.

## 5 CONCLUSION

More than 92% of our patients had at least one modifiable risk factor, with hypertension and diabetes being at the forefront. One out of four patients has become functionally independent at discharge, while hospital mortality was lower than in other Eastern European countries. This indicates an urgent need for targeted risk factor management, acute‐care development, and post‐stroke therapeutic coordination. This analysis provides the most recent data on stroke epidemiological features in Tuzla Canton, B&H. Epidemiological studies, conjoined by other data‐acquisition techniques on stroke patterns and features, will provide a more comprehensive understanding of stroke occurrence, together with its specificities across different subgroups. The overarching goal should be steered toward the development of a national stroke registry, which should be used as a reference for all further stroke management activities.

## FUNDING

The author(s) received no financial support for the research, authorship, and/or publication of this article.

## CONFLICT OF INTEREST

The authors declare that the research was conducted in the absence of any commercial or financial relationships that could be construed as a potential conflict of interest.

## AUTHOR CONTRIBUTION

Conceptualization: Adnan Mujanovic, Dzevdet Smajlovic.

Data Curation: Adnan Mujanovic.

Formal Analysis: Adnan Mujanovic.

Investigation: Adnan Mujanovic.

Methodology: Adnan Mujanovic, Dzevdet Smajlovic.

Project Administration: Dzevdet Smajlovic.

Supervision: Dzevdet Smajlovic.

Writing—Original Draft Preparation: Adnan Mujanovic.

Writing—Review & Editing: Adnan Mujanovic, Dzevdet Smajlovic.

All authors have read and approved the final version of the manuscript.

Adnan Mujanovic had full access to all of the data in this study and takes complete responsibility for the integrity of the data and the accuracy of the data analysis.

## TRANSPARENCY STATEMENT

Adnan Mujanovic affirms that this manuscript is an honest, accurate, and transparent account of the study being reported; that no important aspects of the study have been omitted; and that any discrepancies from the study as planned (and, if relevant, registered) have been explained.

## Data Availability

The datasets generated and analyzed for the current study are not publicly available due to ethical concerns, but are available from the corresponding author upon reasonable request and after clearance from the ethics committee.
